# Implementing integrated models of care: the importance of the macro-level context

**DOI:** 10.5334/ijic.2247

**Published:** 2015-09-23

**Authors:** Toni Ashton

**Affiliations:** School of Population Health, University of Auckland, Private Bag, 92019 Auckland, New Zealand

**Keywords:** models of care, older adults, implementation, transferability, policy context

## Abstract

Reports of how different countries are responding to the need to develop more integrated health and social care services for older adults can provide useful lessons for other health systems. However an understanding of how the wider structural, political, economic and cultural context affects implementation of these models of care is essential when considering the potential for models to be scaled up or transferred to other jurisdictions.

What are the steps to implementing models of community-based primary health care for older adults with complex health and social care needs? This question is currently being addressed by a team of researchers in New Zealand and Canada. The study (called iCOACH-Implementing Community-based models of care for Older Adults with Complex Health and social needs) will use a case study approach to explore the factors which affect implementation of innovative, integrated models of care. The case studies will be based in three jurisdictions: Ontario, Quebec and New Zealand. By comparing cases both within each of these three jurisdictions as well across jurisdictions we aim to tease out key contextual factors which must be taken into consideration in the scaling up or transfer of models of integrated care for older adults. I am a health economist who is leading the New Zealand arm of the research.

The increase in non-communicable diseases, the ageing of populations and the deficiencies associated with traditional health systems in treating chronic conditions are encouraging many countries to pursue more integrated models of care that meet the array of health and social care needs of older adults who have multiple chronic conditions. This Special Issue provides examples of models of care that have been developed in seven different countries. Publications of this type inevitably encourage commentators to reflect upon lessons that might be drawn from these experiences 1, 2 or to consider whether similar models of care (or components of these models) might be scaled up or transferred to other jurisdictions 3.

When considering what lessons might be learned from innovations and practices in other countries, it is not only the design of the model of care that needs to be considered, we also need to think about the factors that have promoted-or inhibited-implementation of that model 4. The focus of implementation research is on the translation of evidence into policy or practice. Although a relatively new field of enquiry, implementation science is now well recognised as a knowledge area which can make an important contribution towards our understanding of why a model of care may work well in one jurisdiction but not in another, or what factors need to be taken into account when transferring models across jurisdictions.

Many conceptual frameworks have been developed in an effort to explain the factors that affect implementation of new services or programmes. However, there now seems to be a growing consensus that a multi-level framework that identifies relevant factors at the macro-(external context), miso-(organisational) and micro-(provider/patient) levels is most appropriate 5, 6. Most attention has been paid to factors at the organisational level (such as workforce capacity, leadership and other variables which affect organisational readiness) or at the provider level (such as the degree of cooperation, multidisciplinary teamwork and information sharing) 5. Less attention has been paid to patient-level variables or to the wider social, cultural, political and economic context in which implementation of new models of care takes place.

In a systematic review of studies reporting measures designed to assess variables that predict the implementation of innovative models of health care, Chaudoir et al. 5 found that only 5 out of 62 measurement instruments included macro-level contextual or structural constructs. This seems surprising, given the potential influence that external contextual factors can have on the successful implementation of new models of care. Mays and Smith 1, for example, when reflecting upon what lessons might be learned from an integrated model of care in Canterbury, New Zealand, suggested that the key factors that had assisted successful implementation were the relative stability of the wider health system, the existence of organised general practice and district-wide cooperation in developing appropriate mechanisms for governance, funding and information sharing.

So what type of macro-level contextual factors might potentially promote or inhibit implementation of integrated models of care? First and foremost are the broad institutional arrangements that give shape to a health system. These include the overall financing, structure and governance of the health system, the roles and responsibilities of key agencies, monitoring and accountability arrangements, and any high level quality control or quality improvement mechanisms. The strategic direction of the health system is also important, including whether or not a country has a clearly articulated strategy for improving service integration. In New Zealand for example, improved integration of services which provide more seamless care for patients is one of five principles which underpin the government's health policy 7.

In the case of services for older adults with complex needs, policies which influence the funding and provision of both formal and informal social care-particularly services provided in the home-are equally as important as policies which relate specifically to the health care system. The availability and coordination of social welfare benefits, housing, pensions and transport policies may also impact upon what services can be provided, by whom and how, and upon the implementation and sustainability of these services.

Various aspects of the economic environment must also be considered. As illustrated in the seven case studies in this Special Issue, the way that services are funded is key to successful implementation of integrated models of care. While a single funding stream is not essential, the pooling of budgets clearly facilitates integration across providers. Even where funding for a range of services is the responsibility of a single agency, funders often earmark money for specific services, thereby creating ‘funding silos’. Unlike England and many European countries, in New Zealand and Quebec, funding for health and social care is the responsibility of the same funding agency, and yet the provision of seamless and integrated care across the two sectors has proved elusive 8. The need to rely on multiple sources of funds or to overcome funding silos adds an additional layer of complexity and has the potential to inhibit the implementation of integrated models of care. Methods of provider remuneration are also likely to impact upon implementation because payment systems can provide powerful incentives (and sometimes disincentives) for collaboration and teamwork. At a more general level, introducing new models of care is likely to be easier during periods when expenditure on health and social care is increasing than during periods of fiscal constraint.

The regulatory environment also needs to be taken into account when studying the implementation of new models of care. Laws and regulations governing professional competencies and scopes of practice, standards of service and safety are clearly important. However the implementation process may also be affected by more general legislation or regulations. For example, privacy laws can inhibit the sharing of patient records 9 and laws designed to discourage restrictive trade practices may discourage collaboration between service providers 10. By the same token, human rights legislation or agreements may promote the development of services for particular population groups such as older people 11 or indigenous populations 12.

Finally, implementation and the potential transferability of models of integrated care are profoundly affected by the characteristics and culture of the community. This includes the political authority of key stakeholders (including professional and consumer associations), the degree of collaboration or competition amongst funders and/or providers, the value placed upon and opportunities for community consultation and engagement, and the many social norms that influence the behaviour of people within a community.

In summary, examining how different countries are responding to the need to develop more integrated health and social care services for older adults can provide useful lessons for the development of services elsewhere. However an understanding of how the wider structural, political, economic and cultural context has affected implementation of these models of care should be a key element in this process. Most of the articles in this Special Issue do not report specifically on how the macro-level context may have affected the design and implementation of the models of care. This may be because comparative analysis was not the objective of these studies. Even so, I would urge researchers to explore and report on any relevant macro-level influences in future analyses of integrated care models. This will strengthen the evidence base not only of the factors that influence implementation processes and outcomes but of the extent to which developments in one jurisdiction can inform service developments elsewhere.
